# EXPANDING BENEVOLENT AGEISM: MEASURING EXPERIENCES OF OLDER ADULTS

**DOI:** 10.1093/geroni/igz038.3515

**Published:** 2019-11-08

**Authors:** Jennifer F Sublett, Toni L Bisconti

**Affiliations:** The University of Akron, Akron, Ohio, United States

## Abstract

Benevolent ageism has recently been recognized as a form of patronizing treatment that older adults experience because of the kind and incompetent age stereotype proposed by the Stereotype Content Model. However, there is limited research that examines older adults’ experiences with patronizing treatment. The aim of this study was to conceptualize benevolent ageism based on older adults’ experiences with items from an existing measure of ageism, the Ambivalent Ageism Scale, and additional items created by us that expand the measurement of benevolent ageist behaviors. In an internet-based sample of older adults who were 65 years old and older (N =135), the benevolent subscale of the Ambivalent Ageism Scale with our additional 10 items demonstrated excellent reliability (α = .90). An exploratory factor analysis cleanly yielded a 4-factor solution that mirrored previous findings, (1) hostile ageism, (2) unwanted help, (3) cognitive assistance/protection, while introducing a new factor of (4) condescending endearment. The findings from this study have widened the scope with which ageism is viewed by examining older adults’ experiences with ageism and conceptualizing characteristics of benevolence that older adults may face due to the widespread belief that they are kind and incompetent. The validation of a scale measuring individuals’ experiences with ageism will provide insight as to whether older adults experience ageist behaviors that people report endorsing and if older adults receive unnecessary offers of help. A recipient’s perspective of ageism will aid in the understanding of the insidious and benevolent characteristics of ageism within society.

